# Anaerobic Solid-State Fermentation of Soybean Meal With *Bacillus* sp. to Improve Nutritional Quality

**DOI:** 10.3389/fnut.2021.706977

**Published:** 2021-08-19

**Authors:** Yanhui Yao, Hongya Li, Jia Li, Baocheng Zhu, Tongguo Gao

**Affiliations:** College of Life Sciences, Hebei Agricultural University, Baoding, China

**Keywords:** *Bacillus*, soybean meal, anti-nutritional factor, bacterial community structure, anaerobic solid-state fermentation

## Abstract

The study evaluated the impact of fermentation with *Bacillus* sp. on the nutritional quality of soybean meal (SBM) and the changes of bacterial community structure during fermentation. High protease-producing strains were screened to degrade SBM macromolecular protein and anti-nutritional factors (ANFs). Unsterilized SBM then underwent an anaerobic solid-state fermentation method to evaluate the effects of fermentation. Results showed that for the nine high-producing protease strains that were screened, acid-soluble protein (ASP) contents in fermented SBM increased, with the highest value found to be 13.48%, which was fermented using strain N-11. N-11 was identified as *Bacillus subtilis*. N-11 fermentation reduced ANFs such as glycinin and β-conglycinin by 82.38 and 88.32%, respectively. During N-11 fermentation, the bacterial richness and diversity in SBM increased but not significantly. The high-yield protease strain *B. subtilis* N-11 selected in this experiment improved the nutritional quality of SBM through fermentation, and it can be used for industrial large-scale production.

## Introduction

Soybean meal (SBM) is a by-product of soybean oil extraction, with high protein content and balanced amino acid composition. It is the most important vegetable protein source in the animal feed industry. However, macromolecular protein and anti-nutritional factors (ANFs) contained in SBM decrease digestibility and absorption, particularly for young animals (1). Residual nitrogen is excreted with feces and pollutes the environment. Therefore, improving the digestion and absorption of SBM by animals has always been a topic of concern.

Microbial fermentation is an effective method to degrade macromolecular protein and ANF compounds to enhance the nutritional quality of the SBM (2). The beneficial effects of feeding fermented SBM (FSBM)-based diets to a variety of ruminants as well as non-ruminants, both young and adult, have also been investigated and established (3, 4). A wide range of microorganisms, such as *Bacillus subtilis, B. licheniformis, Yeast, Lactobacillus, Aspergillus niger*, and *A. oryzae* can be used in FSBM, and the characteristics of FSBM depend on the type of microorganisms (4). Teng et al. (5) used *A. oryzae* and *B. subtilis* to ferment SBM, which increased crude protein content by 0.34 and 8.37% and increased small-sized (<15 kD) proteins by 30 and 58%, respectively. Recently, the *Bacillus* sp. has become a strain of interest based on its characteristics and positive fermentation effects (6).

Solid-state fermentation (SSF) is an effective way for *Bacillus* sp. to degrade ANFs and macromolecular protein (7). Most of the SBM subjected to SSF adopts aerobic fermentation method using sterilized SBM, which can shorten the fermentation time, but increase material loss and cost, and is subject to pollution by *A. flavus* (8). Anaerobic solid fermentation using unsterilized SBM can prevent the previously mentioned limitations; however, there is no information on the nutritional effects and the changes of bacterial communities during SSF by using anaerobic SSF on SBM.

The improvement of nutritional properties of SBM depends on the hydrolysis reaction of protease (4). As a result, the selection of *Bacillus* isolates to ferment SBM was based on their protease activities. In this study, high protease-producing strains were selected to ferment unsterilized SBM using anaerobic SSF, and we evaluated the changes in nutritional quality and bacterial community structure of FSBM.

## Materials and Methods

### Chemicals, Reagents, and Strains

Defatted SBM was purchased from Shandong Koufu Oils & Grains Co., Ltd. (Binzhou, China). Folin–phenol, phytic acid, sulfosalicylic acid, cetyltrimethylammonium bromide, sodium dodecyl sulfate, amylase, and ethylenediaminetetraacetic acid were purchased from Sigma–Aldrich (St. Louis, MO, United States). All other chemicals were of analytical grade locally.

*Bacillus* sp. strains named: B-1, B-2, B-3, B-4, B-5, B-6, B-7, B-8, B-9, B-10, B-11, G4, M1, M-2, M3, M4, M10, 46, W-18, Lipro-1, Lipro-2, J-4, DB-7-6, ED-3-7, Y4, N-2, 2-27, N-11, 20, and 57 were isolated and stored in our laboratory. All strains were microscopically examined using Gram- and Schaeffer–Fulton stains to confirm that they were *Bacillus* spp.

### High Protease-Producing Strains Screening

Primary screening of high protease-producing strains utilized skimmed milk powder as the sole carbon source, as described by Guo et al. (9). The ability of 30 *Bacillus* strains to produce neutral protease and acid protease was tested using the Folin–phenol method as a second screening method (10). Strains were separately cultured in Nutrient Broth medium (5 g/L peptone, 3 g/L of beef extract, 5 g/L of NaCl, 1 l of distilled water, pH 7.0–7.2) at 37°C with shaking at 180 rpm for 24 h. After centrifugation at 8,000 rpm for 15 min at 4°C, the supernatant was obtained as a crude enzyme. One unit of protease activity was defined as the amount of protease required to produce 1 μg tyrosine released from casein hydrolyzed under described conditions for 1 min.

### SBM Solid-State Fermentation

The unsterilized SSF medium (SBM 88.0%, cornmeal 10.0% (NH_4_)_2_SO_4_ 2.0%) was inoculated with 10^9^ CFU/kg *Bacillus* culture, mixed with an equal volume of water, then put into buckets (6 kg), compacted and sealed, and fermented at room temperature for 14 days.

### Chemical Composition of FSBM

Crude protein was measured using the Kjeldahl method with the automatic Kjeldahl apparatus (K9840, Haineng, China). The trichloroacetic acid-soluble protein (TCA-SP) of the sample was determined using the methods described by Ovissipour et al. (11). Glycinin, β-conglycinin, and trypsin inhibitor (TI) were analyzed using a commercially available ELISA assay kit (Longkefangzhou Bio-Engineering Technology Company, Beijing, China). The ferric chloride colorimetric method was used to determine the content of phytic acid (12). The determination of total urease activity was performed according to the Chinese National Standard (GB/T 8622-2006, China) (13). Neutral detergent fiber (NDF) content, acid detergent fiber (ADF) content, ash content, and the pH of SBM and FSBM were determined according to the methods described by Van-Soest et al. (14).

### Strain Identification

Genomic DNA was extracted using the *Easypure* Bacteria DNA kit according to the protocol outlined in the manual (Transgene Biotech Corporation, Beijing). An ~1,500 bp of 16S ribosomal RNA fragment was amplified using forward primer (5′-AGAGTTTGATCCTGGCTCAG-3′) and reverse primer (5′-CTACGGCTACCTTGTTACGA-3′). The 20 μl PCR reaction mixture contained 10× *EasyTaq* buffer (2 μL), 2.5 mM dNTPs (1.6 μl), 5 U/μl EasyTaq DNA Polymerase (0.2 μl), 20 μM primers (1 μL each), crude DNA template (1 μl), and sterile distilled water. Amplification was performed on a thermal cycler with a cycling profile of initial denaturation at 95°C for 5 min; 30 cycles of 95°C for 1 min, 56°C for 1 min, and 72°C for 2 min; and final extension at 72°C for 10 min. The PCR products were sequenced by Huada Gene Technology Co. Ltd. Basic Local Alignment Search Tool (BLAST) services provided by the National Center for Biotechnology Information (NCBI, http://www.ncbi.nlm.nih.gov/) were used in this study for comparison of nucleic acid sequences, and suitable strains were selected for subsequent experiments (15).

### High-Throughput Sequencing of 16S rRNA Gene

According to the result of acid-soluble protein (ASP), the best fermentation group and the control group were sampled to compare the bacterial communities by high-throughput sequencing of 16S rRNA genes. Genomic DNA was extracted using the E.Z.N.A. Soil DNA Kit (Omega Biotek, United States) following instructions outlined in the manual. The 16S rRNA V3+V4 region was amplified with the following primers: forward primer 341 F: 5′-CCTAYGGGRBGCASCAG-3′ and reverse primer 806 R: 5′-GGACTACHVGGGTWTCTAAT-3′. For the bacterial DNA amplification, the reaction was performed in a final volume of 20 μl containing 10 ng template DNA, 4 μl 5× FastPfu Buffer, 2 μl 2.5 mM dNTP, 1.0 U of Taq Polymerase, 0.8 μl of each primer of 5 μM, 0.2 μl BSA, and distilled water. The PCR condition included initial denaturation at 95°C for 3 min; 27 cycles of 95°C for 30 s, 55°C for 30 s, and 72°C for 45 s; and final extension at 72°C for 10 min. The PCR products were sequenced by YUEWEI Co. Ltd. (Beijing, China) using Illumina HiSeq 2500.

### Sequence Analysis

The sequences with high quality were clustered into different operational taxonomic units (OTUs) based on a 97% sequence similarity using the Qiime (V1.9.1) (16) software, and they were aligned with the GreenGenes reference gene database. Alpha diversity (the rarefaction, Chao1 richness, Shannon, and Simpson diversity indices) was used to estimate the biodiversity of bacteria in a single sample (17). Beta diversity analysis was performed to assess the distribution and content of bacteria and evaluate the total diversity in different samples based on the bacterial profile.

### Statistical Analysis

All experiments were performed in triplicate. The data were expressed as mean ± SD. Statistical analysis was done using one-way ANOVA and Duncan's test at a value of 0.05 using SPSS (SPSS Inc., United States).

## Results

### Strain Screening

**Figure 1:**
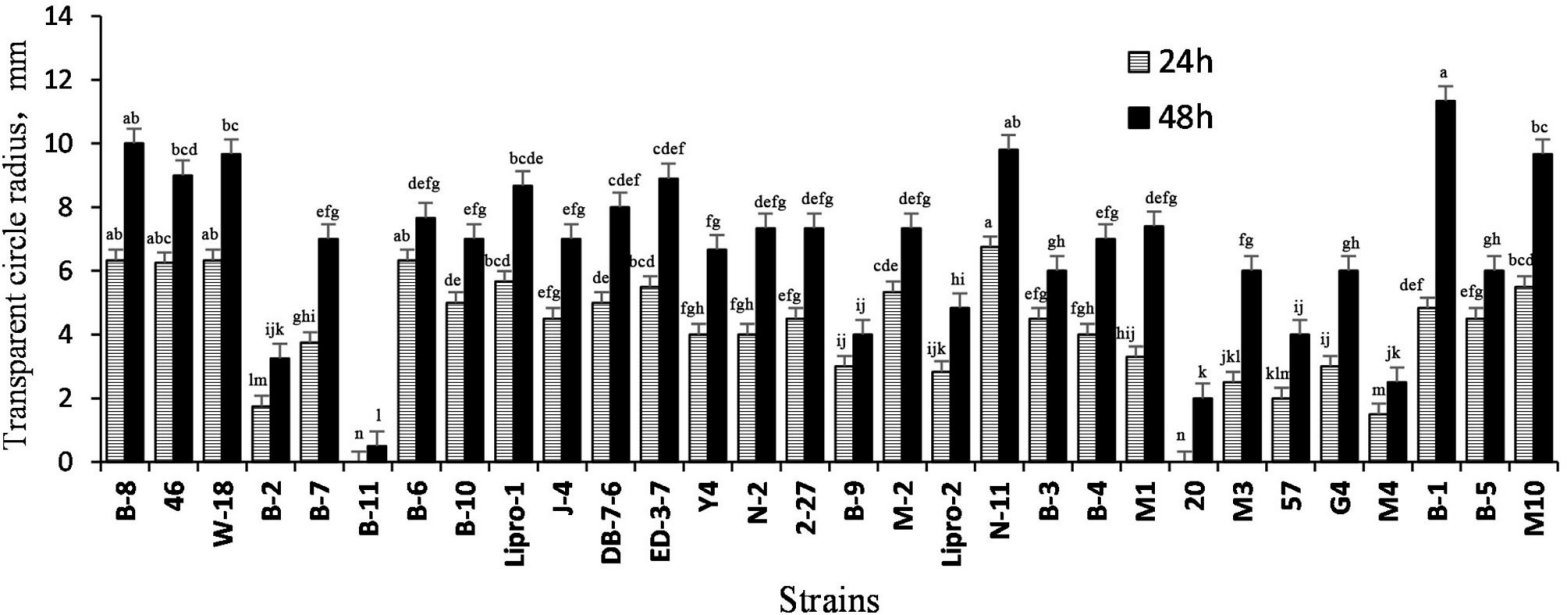
The distance from the edge of the colony to the edge of the transparent circle on skimmed milk plate. Values are expressed as averages ± SD. Different letters indicate significant differences (*P* < 0.05) among groups.

The ability of *Bacillus* strains to degrade skimmed milk during initial screening, where the radiuses of transparent circles were measured, is shown in Figure 1. The radiuses of the transparent circle cultured for 48 h were larger than that of 24 h, and strains B-1, B-8, N-11, M10, W-18, 46, ED-3-7, Lipro-1, and DB-7-6 also generated larger radiuses.

**Figure 2:**
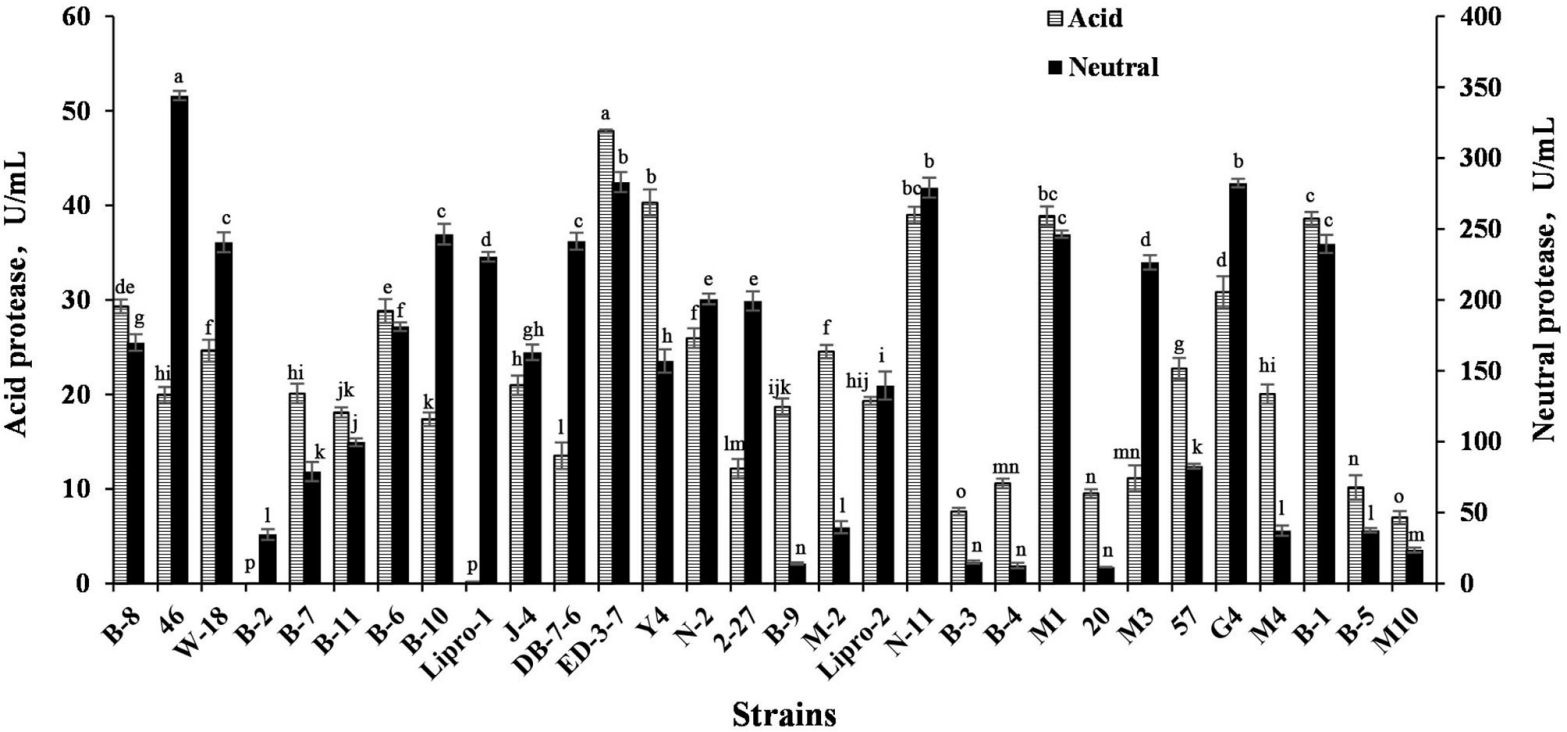
Acid protease and neutral protease production ability of strains. Values are expressed as averages ± SD. Different letters indicate significant differences (*P* < 0.05) among groups.

The acid protease activity and neutral protease activity of 30 strains were tested, with results shown in Figure 2. The acid protease activity of strains ED-3-7, M1, Y-4, B-1, G4, B-8, B-6, N-11, and N-2 was higher than 25 U/mL. The neutral protease activity of 46, ED-3-7, G4, N-11, B-10, M1, DB-7-6, and W-18 was higher than 240 U/mL. According to the above results, nine strains named B-8, 46, ED-3-7, Y-4, N-11, M1, G4, B-1, and W-18 with high protease-producing capacity were chosen for SBM fermentation.

### Changes in Nutrients After Fermentation

**Figure 3:**
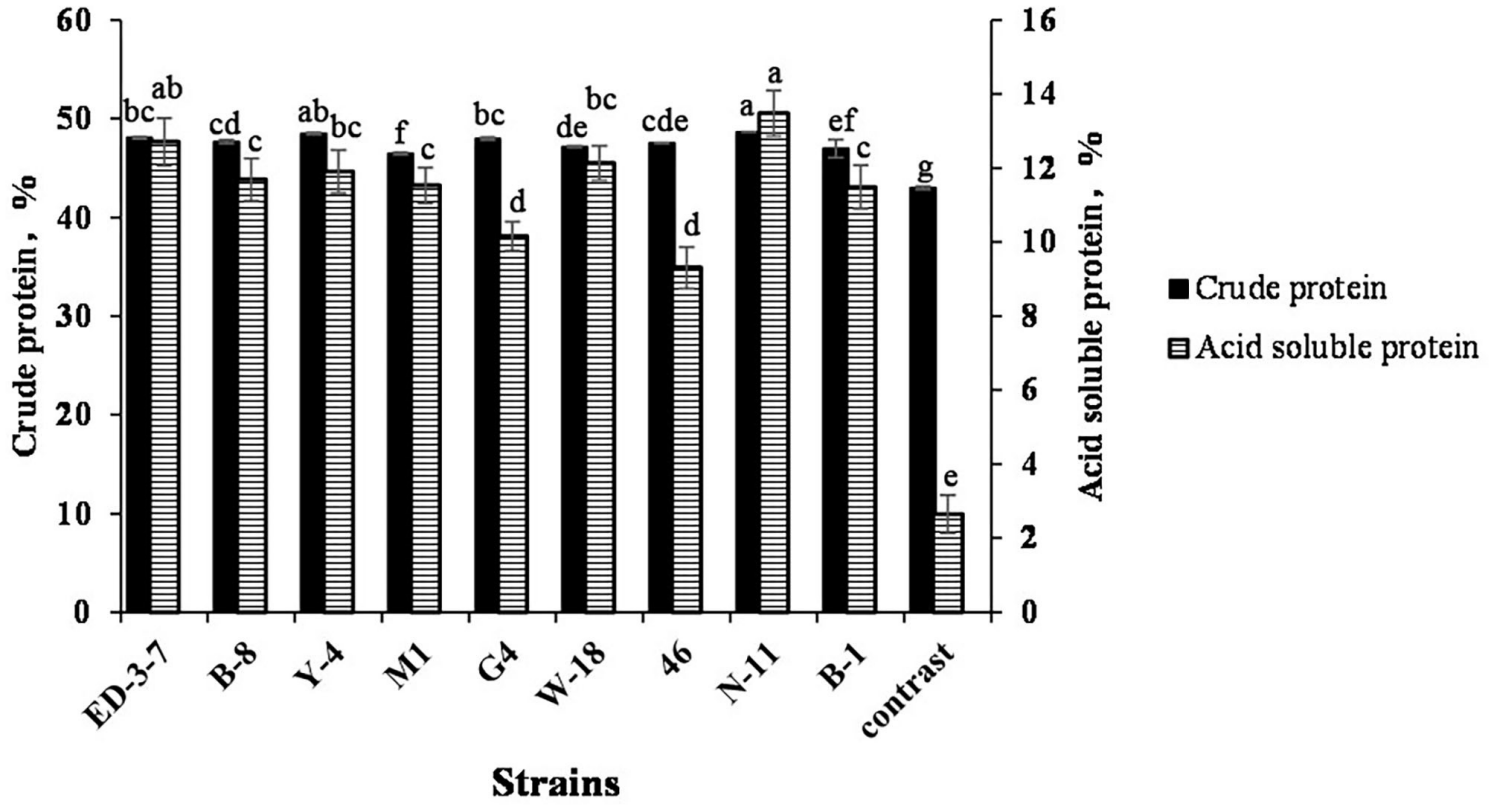
Crude protein and ASP of soybean meal (SBM) and SBM fermented with N-11 (FSBM). Values are expressed as averages ± standard deviation. Different letters indicate significant differences (*P* < 0.05) among groups.

The crude protein content and ASP of FSBM fermented by nine strains are shown in Figure 3. The crude protein content of FSBM reached 46–49% after fermentation. FSBM fermented by N-11, Y-4, and ED-3-7 had a higher crude protein content, increasing by 13.16, 12.77, and 11.81%, respectively, when compared with SBM. ASP contents of FSBM reached 9–14% after fermentation. FSBM fermented with N-11, ED-3-7, and W-18 had a higher ASP content at 13.48, 12.71, and 12.14%, respectively, which was 5.09, 4.80, and 4.58 times that of SBM. ASP accounted for 27.7, 26.48, and 25.74% of crude protein content in FSBM fermented with N-11, ED-3-7, and W-18 strains.

### Strain Identification

After fermentation, N-11 demonstrated improvements in ASP content. Based on morphological, biochemical characteristics, and 16S rRNA gene sequences, N-11 was identified as *B. subtilis*. The 16S rRNA gene sequence was submitted to the GenBank database (accession number: MW345828).

### Chemical Composition of FSBM

Anti-nutritional factors, chemical composition, and hygienic indicators in SBM and FSBM are shown in Table 1. Glycinin, β-conglycinin, urease, and phytic acid decreased significantly by 82.38, 88.32, 93.10, and 72.09%, respectively (*P* < 0.05), compared with those of SBM. TI content in FSBM was <0.3 mg/g according to the ELISA assay kit.

**Table 1:** Anti-nutritional factors (ANFs), chemical composition, and hygienic indicators in SBM and FSBM.

	**SBM**	**FSBM (N-11)**
ANFs		
Glycinin (mg/g)	88.15 ± 0.43a	15.53 ± 0.24b
β-conglycinin (mg/g)	92.78 ± 0.03a	10.84 ± 0.05b
Trypsin inhibitor (mg/g)	23.63 ± 0.12	<0.3
Urease (U/g)	0.29 ± 0.01a	0.02 ± 0.00b
Phytic acid (%)	2.15 ± 0.04a	0.60 ± 0.02b
Chemical composition		
Lipid (%)	1.10 ± 0.01a	0.80 ± 0.01b
Total acid (g/kg)^*^	2.87 ± 0.14b	13.44 ± 0.21a
Crude fiber (%)	9.05 ± 0.04a	6.55 ± 0.05b
Neutral detergent fiber (%)	7.90 ± 0.01a	7.40 ± 0.03b
Acid detergent fiber (%)	11.53 ± 0.03a	11.47 ± 0.03a
Ash (%)	6.46 ± 0.04a	6.09 ± 0.06b
pH^*^	6.51 ± 0.02a	4.99 ± 0.03b
Water content (%)^*^	9.00 ± 0.06b	45.88 ± 0.57a
Hygienic indicator^*^		
Aflatoxin (μg/kg)	3.34	–
Coliform bacteria (MPN/g)	<0.3	<0.3

*SBM, soybean meal; FSBM, soybean meal fermented with N-11*;

* *wet basis; MPN, most probable number; means in a raw with different letters were significantly different (p < 0.05)*.

Lipid, crude fiber, neutral detergent fiber, and ash in FSBM decreased significantly by 27.27, 27.62, 6.33, and 5.73%, respectively, compared with SBM. Total acid in FSBM increased by 368.29% compared with that of SBM, and acid detergent fiber did not change significantly. The pH was 4.99 in FSBM and 6.51 in SBM. Aflatoxin was 3.34 μg/kg in SBM, but not detected in FSBM. Coliform bacteria in both samples was <0.3 MPN/g.

### Changes in Flora After Fermentation With N-11

Samples of SBM (CK) and FSBM fermented by N-11 (T group) were sent to the sequencing company to detect the change of bacterial structure during fermentation.

#### Sequencing Data and Cluster Analysis

**Figure 4:**
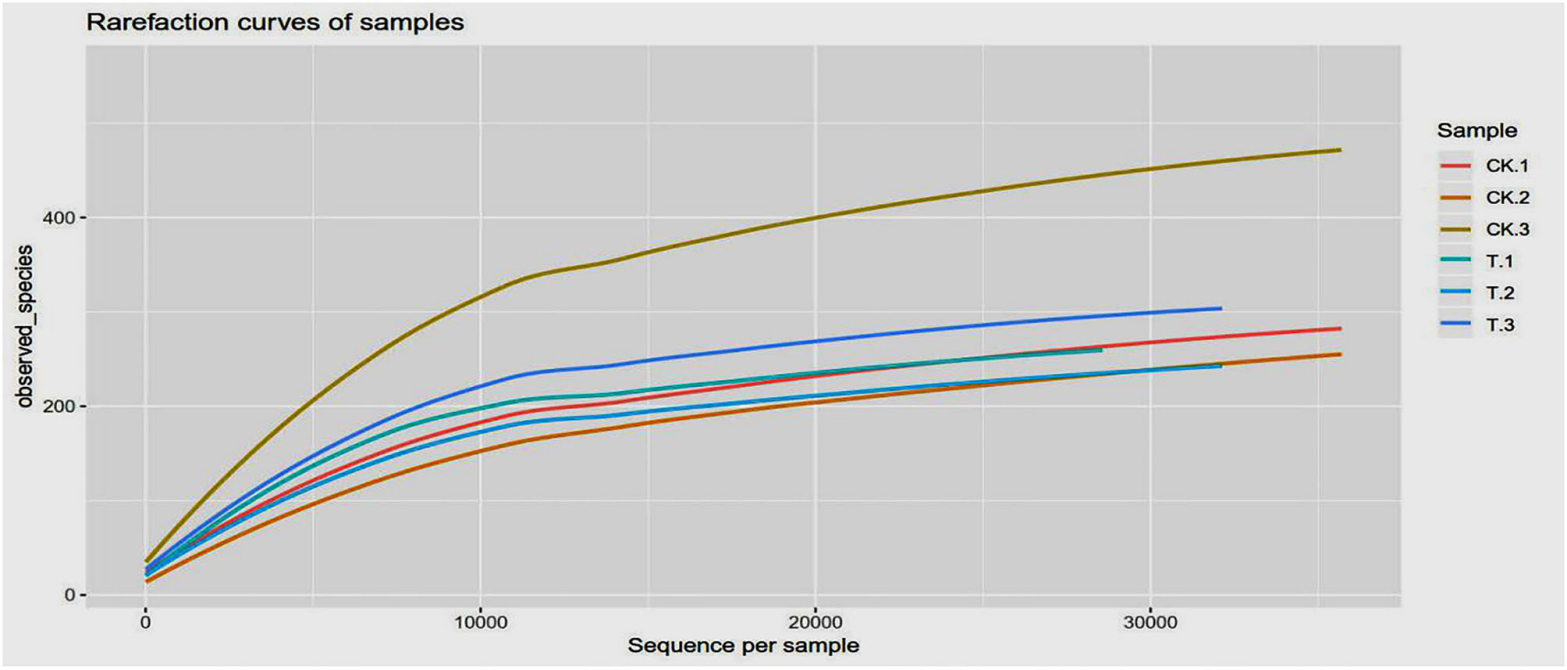
Rarefaction curves based on observed species value. The rarefaction curve was plotted where the X-axis represents the number of clones (sequences) and the Y-axis represents the number of observed species (CK, SBM; T, FSBM).

Figure 4 shows the rarefaction curves of SBM samples. It can be seen that curves gradually plateaued, becoming parallel to the X-axis, indicating that the depth of sequencing had included all species in the sample. Table 2 shows that coverage was more than 99%, which indicates that most of the information of the samples has been obtained from the sequencing data in the current state, and the sequencing meets the analysis requirements.

**Table 2:** Samples alpha diversity analysis index.

**Sample**	**Shannon**	**Simpson**	**PD_whole_tree**	**Goods_coverage**	**Chao1**	**Observed_species**
CK.1	3.448	0.717	15.170	0.998	457.500	321
CK.2	1.998	0.488	15.669	0.998	415.727	294
CK.3	3.639	0.713	19.515	0.998	603.000	516
T.1	3.243	0.727	9.513	0.998	307.558	265
T.2	2.608	0.662	9.142	0.998	300.824	247
T.3	3.596	0.774	10.299	0.998	399.000	309

*CK, soybean meal (SBM); T, soybean meal fermented with N-11 (FSBM)*.

#### Bacterial Alpha Diversity Analysis

Alpha diversity is used to analyze the diversity of the microbial community within-community (18), including Shannon, Simpson, PD values, chao1, and goods coverage. Among them, Shannon, Simpson, and PD values reflect population diversity, and chao1 reflects population richness. The alpha diversity indices of the samples are shown in Table 2. The Shannon index, Chao index, and PD value were larger, and the Simpson index was smaller in the CK group, indicating that the bacteria in the CK group had a higher abundance and diversity. Table 3 shows the results of the alpha diversity *t*-test (*P* < 0.05) in the T group and CK group. The Shannon and Simpson indices of the T group were higher than that of the CK group, indicating that the SBM bacterial diversity after fermentation with N-11 strain increased, but not significant. N-11 fermentation did not significantly change the diversity of SBM bacteria. The chao1 of the T group was lower than that of the CK group, but it did not reach a significant level, indicating that the SBM bacterial richness after fermentation by the N-11 strain did not change significantly.

**Table 3:** Differences in samples alpha diversity.

**Sample**	**Shannon**	**Simpson**	**PD_whole_tree**	**Goods_coverage**	**Chao1**	**Observed_species**
CK	3.028 ± 0.52	0.639 ± 0.08	16.785 ± 1.37	0.998 ± 0.00	492.076 ± 56.75	377.000 ± 28.71
T	3.149 ± 0.29	0.721 ± 0.03	9.651 ± 0.34	0.998 ± 0.00	335.794 ± 31.66	273.667 ± 18.41
*P-*value	0.849	0.376	0.007	0.716	0.074	0.226

*CK, soybean meal (SBM); T, soybean meal fermented with N-11 (FSBM)*.

#### OTU-Specific Analysis

**Figure 5:**
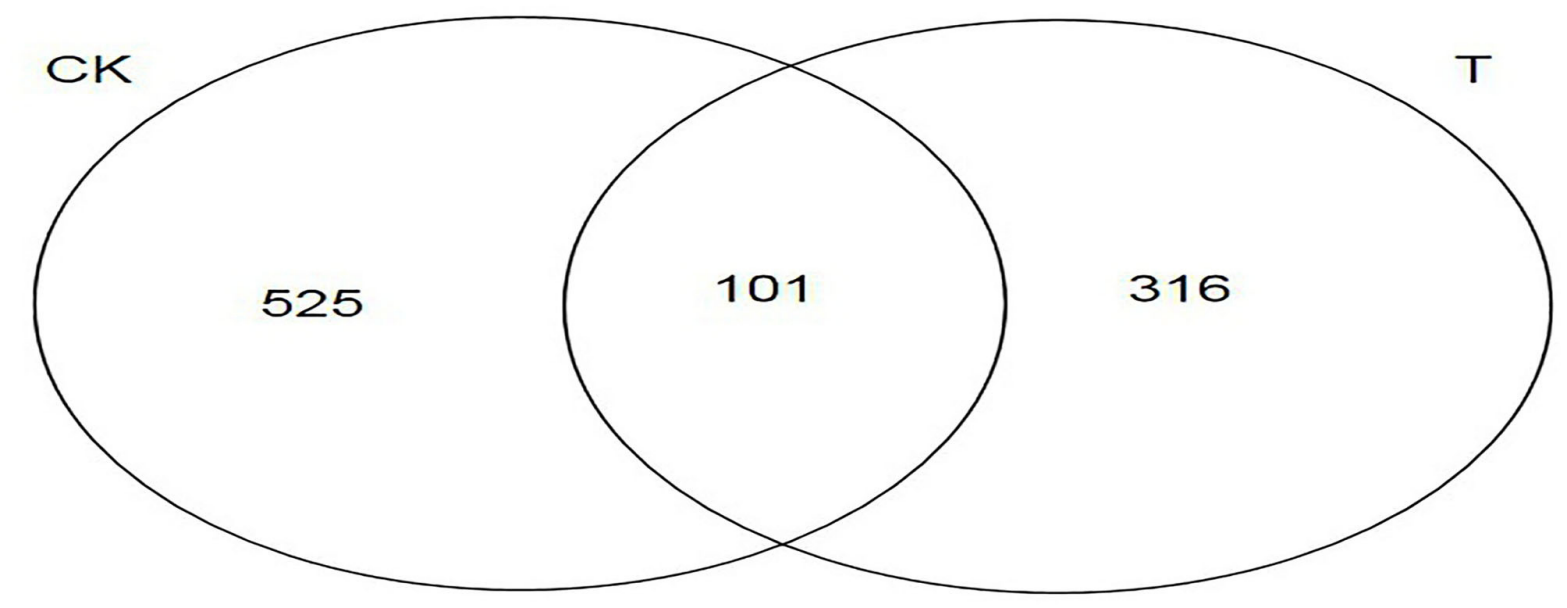
Venn diagram analysis based on operational taxonomic units (OTUs) for different groups. The number of overlapping parts is the number of OTUs shared between the two samples (CK, SBM; T, FSBM).

A Venn diagram can directly show the number, similarity, and specificity of OTUs among groups. It can be seen from Figure 5 that there were 942 OTUs in the two groups. There were 525 OTUs of specific bacteria in the CK group, accounting for 55.73%, 316 OTUs of specific bacteria in the T group, accounting for 33.55%, and 101 OTUs of the same bacteria in the two groups, accounting for 10.72%.

#### Analysis of Community Structure at the Gate Level

**Figure 6:**
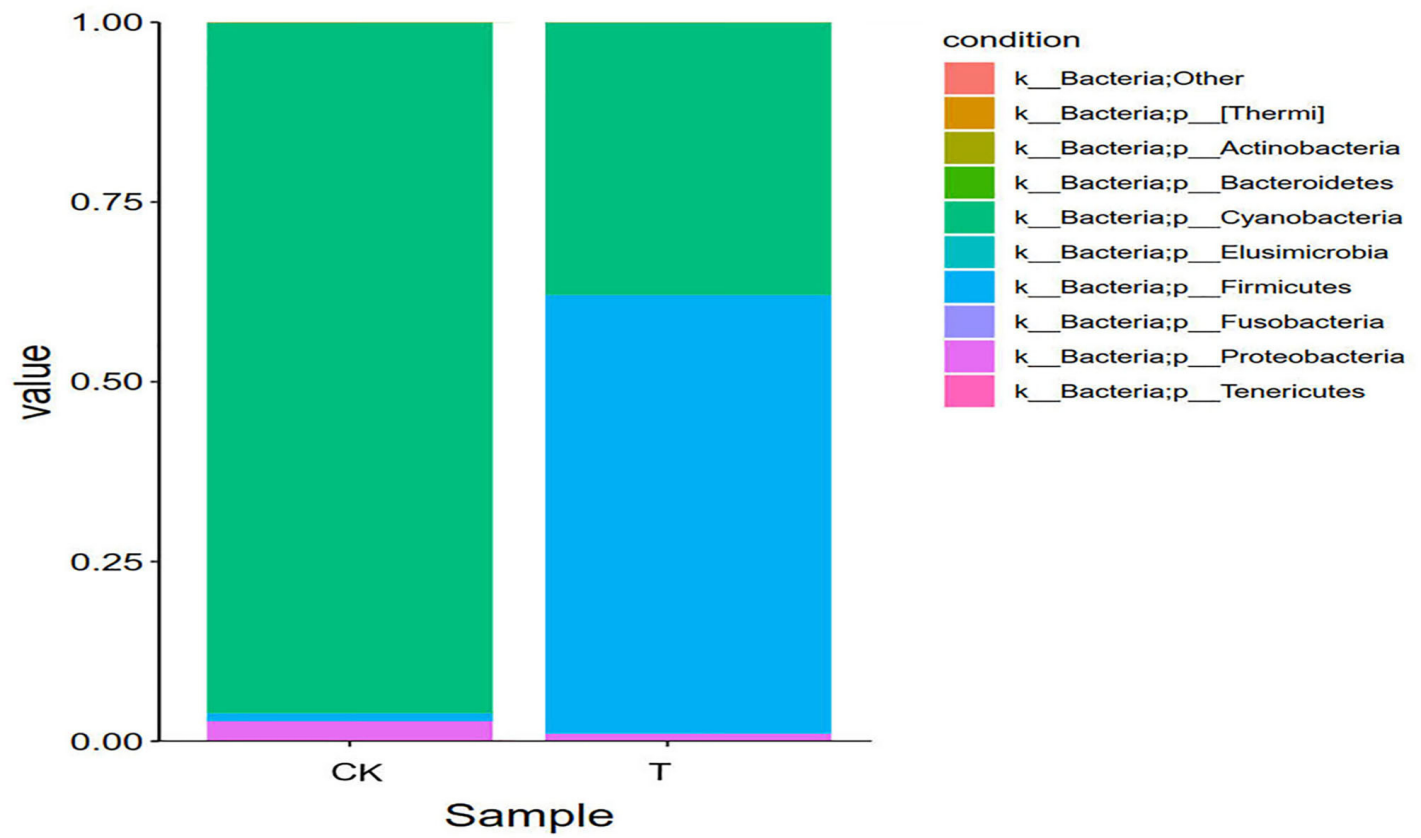
Relative abundance of species at the phylum level. The abundance is represented in terms of the percentage of the total effective bacterial sequences in the sample. The top 10 abound taxa are shown (CK, SBM; T, FSBM).

The sequences are classified according to the reference taxonomy in the reference database. The species classification unit is divided into five layers, which are Phylum, Class, Order, Family, and Genus. The microorganisms identified in the six samples were bacteria. Figure 6 is a histogram corresponding to the profiling of each sample at the gate level, which showed species that were abundant and their proportion in each sample.

It can be seen from Figure 6 that at phylum level, the first eight dominant phyla in the six samples were Cyanobacteria, Firmicutes, Proteobacteria, Bacteroidetes, Actinobacteria, Fusobacteria, Tenerictes, and Elusmicrobia. In the CK group, Cyanobacteria was the dominant bacteria (96.02%), the proportion of Cyanobacteria in SBM decreased, and Firmicutes increased. The relative abundance of Cyanophyta and Firmicutes in FSBM was 37.90 and 61.02%, respectively.

At the genus level, *Lupinus* was the dominant genus of SBM, and *Bacillus* was the dominant genus of FSBM, with a relative abundance of 56.49%.

#### Beta Diversity Analysis

**Figure 7:**
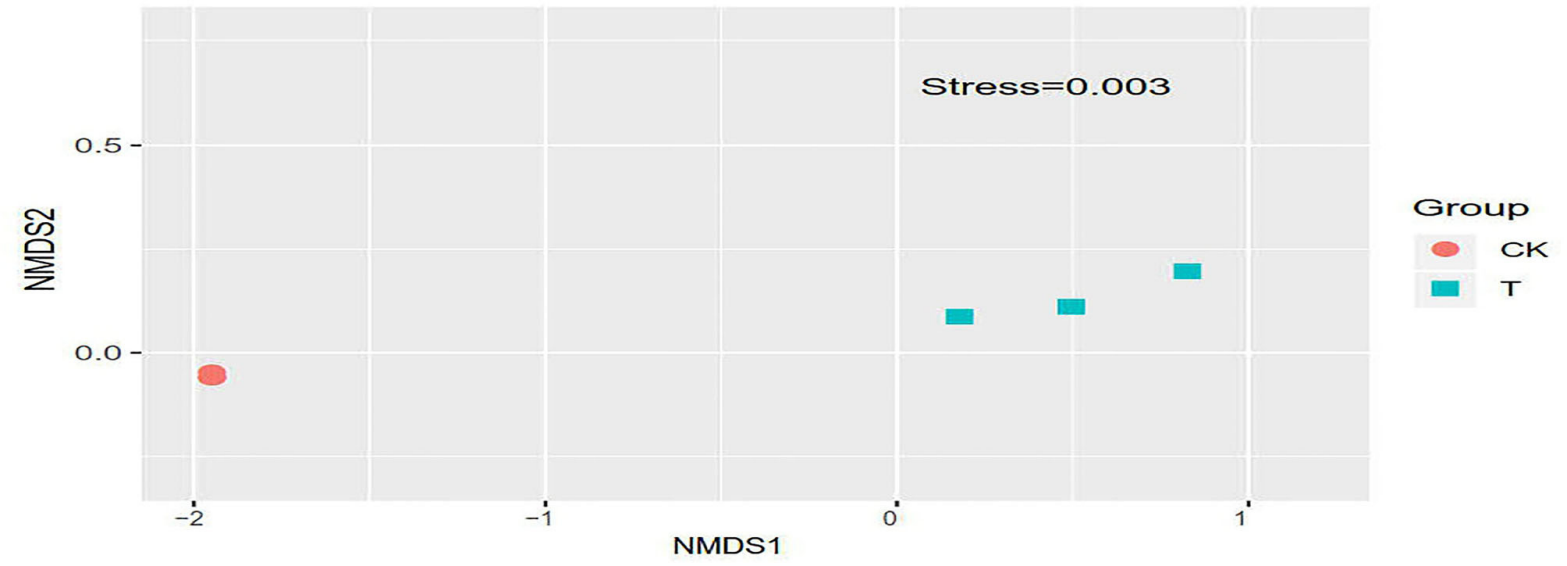
Non-Metric Multi-Dimensional Scaling plot based on weighted UniFrac distance. In each sample name, CK and T represent the sample from SBM and FSBM, respectively. Points with the same pattern represent the three replicate samples from the same group.

Non-metric Multidimensional Scaling Analysis (NMDS) reflects the degree of difference between samples using the distance between points. The results showed that the difference between the groups was greater than the difference within the groups, indicating that the grouping was meaningful. It can be seen from Figure 7 that the difference in the bacterial communities of the three T groups was relatively large compared with that of the CK groups, and the gap between the samples is farther, indicating that the bacterial community of the T groups changed significantly, and the CK groups had a smaller change.

## Discussion

### Effect of Fermentation Method on the Quality of FSBM

Aerobic SSF (ASSF) is a common method used in SBM fermentation (19, 20). The two-stage (first stage, aerobic fermentation; second stage, anaerobic fermentation) SSF process with different microorganisms has also been studied in recent years (21, 22). Anaerobic SSF has unique advantages in water and energy savings, decreased weight loss of material, and environmental protection; it will be the future direction of the fermentation industry (23). *Bacillus* spp. a facultative anaerobic bacteria, can grow in anaerobic fermentation conditions (24). As far as we know, all *Bacillus* sp. reported in SBM fermentation were used in ASSF. In this study, anaerobic SSF using *B. subtilis* was attempted to improve SBM quality.

Although the duration of anaerobic fermentation is in general much longer than that of the ASSF, the quality of the fermented SBM is very similar in the methods. For example, several studies reported the chemical changes in FSBM after 24 h fermentation by *Bacillus* sp. Zheng et al. (6) found that crude protein and ASP increased to 46.67 and 10.91%, and ANFs, such as glycinin, β-conglycinin, and TI decreased significantly by 86.0, 70.3, and 95.01%, respectively; Liu et al. (25) reported that the levels of glycinin and β-conglycinin were reduced by 78 and 43.07%; Zhang et al. (26) reported that the amount of TCA-SP in FSBM was 4.8-fold higher than SBM and that the glycinin and β-conglycinin decreased by 92.4 and 88.4%. Similar results were achieved after 14 days of anaerobic fermentation in this study, ASP content fermented using N-11 was 5.09 times more than SBM, and glycinin and β-conglycinin decreased significantly by 82.38 and 88.32%, respectively. The increase in ASP was likely due to the hydrolysis of macromolecular proteins. ASP was assumed to consist of small molecular peptides [2–20 amino acid (AA) residues] and free AAs (27), which can be directly absorbed in the animal gut system.

### Key Bacterium Determined FSBM Product

Microbial proteases are key enzymes involved in FSBM, which can hydrolyze peptide bonds in target proteins. The nutritional quality of FSBM can vary depending on the type of microorganism used. *Aspergillus, Lactobacillus*, and *B. subtilis* are the most popular microorganisms due to their capacity to produce enzymes. *B. subtilis* is one of the most commonly used probiotics in the fodder industry because of its safety, high efficacy, low price, and ability to secrete considerable amounts of enzymes, including proteases, lipase, amylase, and carboxypeptidase (28). The quality of FSBM is different, which can be attributed to different metabolic activities and enzymes of strains involved. Wang et al. (19) investigated SSF of SBM using a mixed starter culture of *Streptococcus thermophilus, B. subtilis* MA139, and *Saccharomyces cerevisae*. The lactic acid content increased with increasing initial moisture content, while the pH decreased to 5.0 (19). In this study, the pH of FSBM inoculated by N-11 decreased to 4.99, which may be related to acetic acid produced by *Bacillus* and *Lactobacillus* (29), which were the dominant bacteria during fermentation. A decrease in crude fiber and neutral detergent fiber in FSBM were observed, this may be due to the production of cellulase by *B. subtilis* (30), which is consistent with other reports (22, 31). Lower NDF and phytic acid indicated that FSBM may have a higher nutrient digestibility compared with SBM, which was consistent with previous reports (32).

In recent years, the research of strain genomics has become increasingly important in fermentation and application. A complete genome sequence could correlate the function of strain with the activities of various enzymes expressed in SSF (33). Based on genomics, bioengineering methods were applied to enhance the α-amylase in the aprA-deficient strain of *Bacillus licheniformis* for increasing SBM utilization rate (34). The key enzymes proteases involved in FSBM can be modified to enhance the expression according to genome association analysis (35, 36). Genome research will further improve the quality of SBM fermented by N-11 strain, which is the further research direction.

### Bacterial Communities Changed During Fermentation

In this experiment, high-throughput sequencing of microbial communities in FSBM were analyzed to explore changes as a result of inoculation with N-11. After inoculating N-11, the abundance and diversity of bacterial communities increased. At the phylum level, Firmicutes and Proteobacteria dominated in FSBM, Proteobacteria and Firmicutes were the most common bacteria in the environment (37). Firmicutes have a high content of peptidoglycan in the cell wall, and most of them can produce spores, making them resistant to dehydration and extreme environments. α-Proteobacteria is the main component of Proteobacteria, which can play a role in the carbon and nitrogen cycle in an anaerobic environment. At the genus level, *Lupinus* was the dominant genus in SBM, and *Bacillus* was the dominant genus in FSBM. Similarly, *Bacillus* became the main species after 24 h of natural fermentation under aerobic conditions (20). The result of NMDS analysis in this study showed that the difference of the bacterial communities in the three T groups was larger than that of the CK groups, and the gap between the samples was far, indicating that the bacterial community in the T groups changed significantly. The addition of N-11 strains increased the abundance and diversity of bacteria in fermented SBM, but there was no significant difference.

## Conclusion

In this study, *B. subtilis* N-11 was screened for its ability to improve the nutritional quality of unsterilized SBM using anaerobic SSF, and its impact on the bacterial community in FSBM under this condition. The crude and ASP contents increased noticeably, while TI, urease, and phytic acid contents decreased after fermentation. The diversity and richness of the microbial community increased; *Bacillus* sp. was the dominant bacterium in FSBM. These results suggested that anaerobic SSF with *B. subtilis* can substantially improve the nutritional quality of SBM.

## Data Availability Statement

The datasets presented in this study can be found in online repositories. The names of the repository/repositories and accession number(s) can be found at: https://www.ncbi.nlm.nih.gov/genbank/, MW345828.

## Author Contributions

BZ and TG conceived and designed the experiments. YY and JL determined the physicochemical parameters. YY analyzed the data and wrote the original draft. HL and TG edited and approved the final manuscript. All authors contributed to the article and approved the submitted version.

## Conflict of Interest

The authors declare that the research was conducted in the absence of any commercial or financial relationships that could be construed as a potential conflict of interest.

## Publisher's Note

All claims expressed in this article are solely those of the authors and do not necessarily represent those of their affiliated organizations, or those of the publisher, the editors and the reviewers. Any product that may be evaluated in this article, or claim that may be made by its manufacturer, is not guaranteed or endorsed by the publisher.
